# Self-confidence as a mediator in the relationship between executive functioning and depression among ICU survivors: a latent variable analysis

**DOI:** 10.1186/s13054-024-05136-2

**Published:** 2024-10-26

**Authors:** Elias Johannesson, Johan Malmgren

**Affiliations:** 1 Department of Social and Behavioural Studies, University West, Trollhättan, Sweden; 2 Department of Anaesthesiology and Intensive Care Medicine, Institute of Clinical Sciences, Sahlgrenska Academy, University of Gothenburg and Sahlgrenska University Hospital, Blå Stråket 5, 413 45 Gothenburg, Sweden

**Keywords:** Intensive care, Executive functioning, Depression, Cognitive dysfunction, Self-confidence, Self-esteem, Intensive care recovery, Mediation analysis, Mental health outcomes, Quality of life

## Abstract

**Background:**

Executive dysfunction and depression are common among ICU survivors, yet the mechanisms linking these two factors remain poorly understood. Self-confidence has been implicated as a key mediator in the relationship between cognitive impairments and mental health outcomes. This study aimed to explore the mediating role of self-confidence in the association between executive functioning and depression in ICU survivors.

**Method:**

A provisional questionnaire was used in a cross-sectional study to assess quality of life in 395 adult ICU survivors, each with a minimum 72-h stay at one of three ICUs at a Swedish university hospital, six months to three years post-discharge. Responses to questions on executive function, self-confidence, and depression were analysed. Structural equation modelling and confirmatory factor analysis were employed to examine the mediating effect of self-confidence on the relationship between executive function and depression. Model fit was evaluated using established indicators. Reliability of the measures was assessed using McDonald’s Omega and Cronbach’s Alpha.

**Results:**

A significant positive correlation was found between depressive symptoms and both diminished self-confidence (*r* = 0.80, *p* < 0.001) and poorer executive function (*r* = 0.55, *p* < 0.001). Additionally, a correlation was observed between reduced self-confidence and compromised executive function (*r* = 0.62, *p* < 0.001). Age was negatively associated with depression, self-confidence, and executive function, while male gender was positively correlated with higher self-confidence.

Mediation analysis revealed that the effect of impaired executive function on depressive symptoms was fully mediated by diminished self-confidence (B = 0.45; 95% CI 0.34–0.59). The direct effect of executive function on depression became non-significant when self-confidence was included in the model (B = 0.07, *p* = 0.18), suggesting complete mediation. The overall model fit was satisfactory (CFI = 0.962; RMSEA = 0.075), highlighting the robustness of the mediation pathway.

**Conclusions:**

Self-confidence mediates the relationship between executive function and depression among ICU survivors. Interventions aimed at enhancing self-confidence could mitigate depressive symptoms in the ICU survivor population. Longitudinal studies are needed to confirm these findings and further explore the causal pathways involved.

*Trial registration* ClinicalTrials.gov Ref# NCT02767180; Registered 28 April 2016.

**Supplementary Information:**

The online version contains supplementary material available at 10.1186/s13054-024-05136-2.

## Introduction

Surviving intensive care often leads to significant mental and psychological challenges, including post-traumatic stress disorder (PTSD), anxiety and depression, all of which significantly impact long-term quality of life (QoL) and functional outcomes [1, 2]. Persistence or severity of anxiety and depression, frequently co-existing as comorbidity, may lead to disruption of daily activities, increased suicide risk, and deterioration of health [3–6]. Furthermore, depression may be exacerbated by persistent negative self-perception and difficulties in recognising personal abilities, stemming from low self-confidence. Preventive interventions such as diaries, intended to mitigate psychological effects by aiding in the understanding of the intensive care unit (ICU) period, have shown some effectiveness [7].

Cognitive dysfunction after intensive care, such as difficulties with attention, memory, and executive functions, has been well described [8]. It can lead to long-term cognitive impairment (LTCI), persisting months to years after discharge, and is independently associated with depression in ICU survivors, underscoring the importance of addressing depressive symptoms early in the recovery process to mitigate cognitive decline [9].

The interplay between depression and self-confidence, as detailed by Beck et al., encompasses both emotional and cognitive dimensions, particularly influencing how individuals with low self-confidence process negative thoughts, thereby impacting depression [10]. In ICU survivors, there is a well-documented relationship between self-confidence, depression, and executive function, underscoring the importance of addressing these factors in recovery [11, 12].

This study aims to examine how self-confidence mediates the relationship between executive function and depression in ICU survivors, hypothesising that it could be a key intervention point to reduce depression risk in this group.

## Methodology

### Questionnaire development

The development of a provisional questionnaire assessing quality of life and other burdens after surviving intensive care has been described in detail in previous publications [13, 14]. Briefly, thirty-two adult ICU survivors were purposively sampled from the post-ICU clinic in a general ICU in a Swedish university hospital. They were interviewed with a semi-structured technique at least six months post-discharge, following established methodology on disease-specific questionnaire creation used in oncology [15]. From these interviews, 195 unique issues were extracted and rephrased into questions (Table S1). All questions were categorised into 13 domains: Cognition, Fatigue, Physical Health, Pain, Mental Health, Activities of Daily Living (ADL), Sleep, Appetite and Alcohol, Sexual Health, Sensory Functions, Gastrointestinal Functions, Urinary Tract Functions, and Work Life. These domains were subsequently converted into a comprehensive questionnaire.

### Study design and participants

The questionnaire was employed in a cross-sectional study to compare responses between ICU survivors and a non-ICU-treated control group, which was matched for age and sex and randomly selected from the Swedish Population Registry. Participants included all adult ICU survivors admitted from February 2013 to December 2015 to one of three mixed ICUs at a university hospital in Gothenburg, Sweden. Eligibility required a minimum ICU stay of 72 h. The rationale for choosing 72 h was twofold. First, it was the length of stay after which survivors were offered a visit to the post-ICU clinic. Second, an abundance of patients with very short ICU length of stay risked diluting any long-term findings. Patients with neurological or neurosurgical diagnoses at admission were excluded. The evaluation occurred between six months and three years after ICU discharge. Of the 518 contacted ICU survivors, 395 completed the questionnaire, yielding a response rate of 76.2%. The current study does not aim to compare results between the two groups. Thus, only the ICU survivor group was included.

### Measures

This study examined three primary constructs derived from a provisional questionnaire developed for ICU survivors: self-confidence, executive function, and depression. Self-confidence was assessed using answers from two questions: *Low self-confidence* and *Low self-esteem* (Cronbach's Alpha = 0.89). Executive function was evaluated through the questions *Difficulties in taking initiative*, *Difficulties in prioritising*, and *Difficulties in finding alternative solutions* (McDonald's Omega = 0.87). Depression was measured considering its frequent comorbidity with anxiety, using three questions: *Feeling low-spirited*, *Feeling depressed*, and *Experiencing periods of anxiety* (McDonald's Omega = 0.92).

All constructs used a six-point Likert scale from "Never" to "All the time." Control variables included age, sex, education level, and comorbidity. The high reliability coefficients across all measures support their validity in assessing these psychological constructs in ICU survivors.

A more comprehensive rationale for each construct, including their theoretical foundations and relevance to ICU survivors, is provided in the supplementary materials.

### Data analysis

We employed a multi-step analytical approach to examine the relationships between executive function, self-confidence, and depression. Descriptive statistics characterized the study sample, while bivariate correlations were used to investigate the relationships between depressive symptoms, self-confidence, and executive function. Reliability was measured using McDonald's Omega for constructs with ≥ 3 indicators and Cronbach's alpha for those with ≤ 2 indicators. A threshold of > 0.7 ensured good convergent validity [16].

Model fit indices were employed to assess the degree to which our hypothesized statistical model adequately represented the observed data, thereby validating the structural relationships posited in the theoretical framework. The model fit was evaluated using established indicators of absolute fit, including root mean square error of approximation (RMSEA), comparative fit index (CFI), Tucker-Lewis index (TLI), and standardised root mean square residual (SRMR) [17]. The criteria for determining a well-fitting model followed established benchmarks (CFI ≥ 0.95; SRMR ≤ 0.09; RMSEA ≤ 0.09) [18].

To address missing data, we utilised the Full Information Maximum Likelihood (FIML) method, which integrates missing data management directly into model estimation to provide accurate estimates [19].

The CFA focused on establishing the measurement properties of the latent variables—executive functioning, self-confidence, and depression—by examining the relationships between observed indicators and their respective latent constructs. The SEM analysis explored the mediation model, investigating how self-confidence mediates the relationship between executive functioning and depression.

This mediation part was conducted using bootstrapping with 5,000 iterations to assess the indirect effects robustly. Simultaneous evaluation of the measurement model and testing of the mediation pathway provides a comprehensive understanding of the interplay between executive functioning, self-confidence, and depression, highlighting potential mechanisms underlying the psychological processes involved.

The analysis was conducted in two sequential phases. First, we evaluated the measurement model to ensure a satisfactory fit. Subsequently, we tested our initial hypothesis (H₁) through mediation analysis, employing SEM with maximum likelihood (ML) estimation. This latter phase utilized the sample covariance matrix as input data [20].

We employed a two-stage analytical approach using confirmatory factor analysis (CFA) and structural equation modelling (SEM) to examine our hypothesized relationships.

In the first stage, we used CFA to evaluate the measurement model. This process assessed the relationships between observed indicators and their respective latent constructs (executive functioning, self-confidence, and depression), establishing the measurement properties of these latent variables. We evaluated the model fit to ensure it adequately represented our data.

The second stage involved SEM to test our mediation hypothesis (H₁). This analysis explored how self-confidence mediates the relationship between executive functioning and depression. We employed maximum likelihood estimation with the sample covariance matrix as input. To robustly assess indirect effects, we conducted bootstrapping with 5000 iterations.

Consistent with best practices, we calculated direct and indirect effects, standard errors, and confidence intervals using bootstrapping with 5000 replications [21]. A mediation effect was considered significant if the 95% bias-corrected bootstrap confidence interval (CI) did not include zero.

To address potential multicollinearity due to strong correlations among the independent variables, we assessed the Variance Inflation Factor (VIF), with a VIF value greater than 10 suggesting a need for remediation. In our model, the highest VIF was 1.54, indicating no significant multicollinearity issues [22].

All analyses were conducted using Mplus version 8.9.

## Results

### Descriptive and correlational analyses

The mean age was 62.8 years (range 19–89), with 61% being male. The median ICU stay was 5.5 days (range 3 to 78.6), with a median SAPS score of 59 (range 16–100). A significant majority (78.5%) had undergone mechanical ventilation, with the median duration of ventilation being four days (range 0 to 74 days). The average number of comorbidities was 2.0 (range 0–9), with the most common being hypertension (50%), mental disease (e.g. depression, anxiety; 19%) and heart failure (16%) [13]. Reliability was high for all three constructs (0.87–0.92). Table 1 shows the characteristics of the scales, with all factor loadings from the CFA exceeding 0.84 (Table 1). Furthermore, the CFA indicated a satisfactory fit for the measurement model with a chi‐square value of 44.990 (*p* < 0.001) at 17 degrees of freedom. The remaining fit measures indicated that the model adequately fit the data (CFI = 0.996; RMSEA = 0.057).

**Table 1:** Descriptive statistics for indicators of latent variables (factors) and control variables (DE, reliability and factor loadings

Factor	Abbr	Loading	*M*	*SD*	*SE*	*n*	Reliability
Depression	DE1	0.95	2.63	1.39	0.07	383	0.92*
	DE2	0.96	2.10	1.42	0.07	382	
	DE3	0.87	1.87	1.36	0.07	382	
Poor self-confidence	SC1	0.91	1.97	1.28	0.07	376	0.89^†^
	SC2	0.94	1.91	1.28	0.07	377	
Poor executive function	EF1	0.88	1.89	1.25	0.06	378	0.87*
	EF2	0.88	1.73	1.14	0.07	303	
	EF3	0.85	1.59	1.01	0.06	279	

**Fig. 1:**
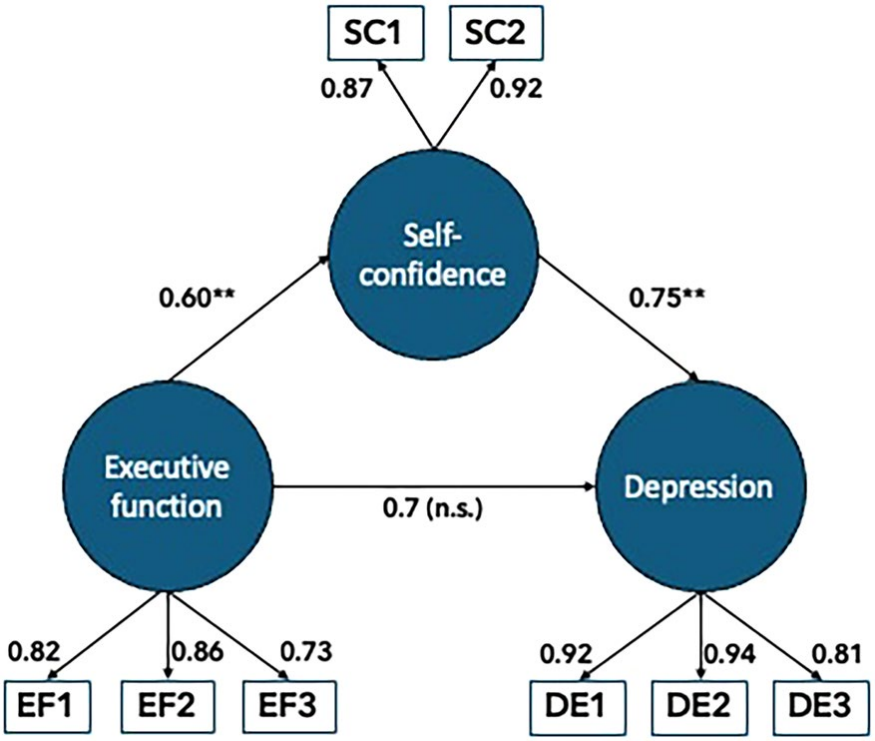
Standardised regression coefficients of the mediating effect of executive function on depression via self-confidence; **p < 0.001. n.s = non-significant

*Abbr.* = Abbreviation for indicators in Fig. 1; DE1 = Feeling low-spirited; DE2 = Feeling depressed; DE3 = Periods of anxiety; SC1 = Self-esteem; SC2 = Self-confidence; EF1 = Difficulties taking initiatives; EF2 = Difficulties prioritising; EF3 = Difficulties finding alternative solutions; *M* = Mean; *SD* = Standard deviation; *SE* = Standard error; *n* = number of participants; *Min* = Minimum value; *Max* = Maximum value. * = McDonald’s Omega. † = Cronbach’s Alpha

Bivariate correlations among the latent variables and control variables are presented in Table 2.

**Table 2:** Correlations between latent variables (1–3) and control variables (4–7)

	1	2	3	4	5	6	7
1. Depression	–						
2. Poor self-confidence	**0.80**	–					
3. Poor executive function	**0.58**	**0.66**	–				
4. Gender	0.08	**0.13**	0.10	–			
5. Age	**− 0.20**	**− 0.22**	**− 0.28**	**− **0.02	–		
6. Educational level	− 0.08	− 0.05	0.07	0.03	− **0.38**	–	
7. Comorbidities	0.02	0.03	− 0.04	0.01	**0.28**	− **0.13**	–

Significant coefficients are shown in bold

There was a significant association between the severity of depressive symptoms and diminished self-confidence, with a strong positive correlation (*r* = 0.80, *p* < 0.001). Similarly, a significant positive correlation was observed between depressive symptoms and poorer executive function (*r* = 0.58, *p* < 0.001), indicating that higher levels of depression are associated with greater challenges in executive function. Additionally, a substantial positive correlation was found between reduced self-confidence and compromised executive function (*r* = 0.66, *p* < 0.001), suggesting that lower self-confidence is linked with poorer executive functioning.

Among the variables controlled for in the study, age was found to have a significant negative relationship with each of the three examined latent variables; older age was associated with reduced levels of depression (*r* = − 0.20, *p* < 0.001), enhanced self-confidence (*r* = − 0.22, *p* < 0.001), and improved executive function (*r* = − 0.28, *p* < 0.001). Furthermore, the analysis identified a gender difference in the correlation with self-confidence, where being male was positively and significantly correlated with higher self-confidence (*r* = 0.13, *p* = 0.020).

A multiple regression analysis, using self-confidence and executive function as predictors of depression, resulted in a less optimal model fit (CFO 0.911; RMSEA 0.114) compared to a mediation analysis (CFI 0.962; RMSEA 0.075).

### Mediation analysis

Figure 1 presents the analyses using self-confidence as a mediator in the relationship between executive function and depression. The pathway from difficulties in executive function to diminished self-confidence was positive (*path a*: *B* = 0.60, *p* < 0.001), indicating that problems with executive function are associated with lower levels of self-confidence. Additionally, the impact of self-confidence on depressive symptoms, while controlling for other covariates (*path b*: *B* = 0.75, *p* < 0.001), was significant. Finally, the adjusted analysis reveals that the direct effect of executive function on depression is non-significant (*path c*: *B* = 0.07, *p* = 0.18), once controlling for gender, age, education level, and comorbidities. Rather, it suggests that the effect is completely mediated, consistent with the results showing a fully mediated model, as stipulated in the initial hypothesis.

Further analysis of the mediation effects showed that the influence mediated by self-confidence was significant (*a* × *b*: *B* = 0.45; 95% CI 0.34–0.59). This suggests that the adverse indirect effects of poor executive function on depression operate through its impact on lowering self-confidence.

## Discussion

In this cross-sectional study of adult ICU survivors, we identified diminished self-confidence as a key mediator in the relationship between impaired executive function and depressive symptoms. This finding underscores the intricate interplay between cognitive impairments and mental health outcomes, highlighting the need for targeted interventions that address both cognitive and emotional challenges in ICU survivors.

Previous research by Duggan et al. has already established the link between executive dysfunction and depression in ICU survivors, showing that impaired executive function three months post-ICU is associated with increased depressive symptoms and poorer mental health-related quality of life (HRQoL) up to 12 months after ICU discharge [11]. Our study builds on this by identifying self-confidence as a critical factor in this relationship, suggesting that enhancing self-confidence could mitigate the negative impact of cognitive impairments on mental health in ICU survivors.

The role of self-confidence in attenuating depression is supported by existing literature. In a multicentre RCT by Horrell et al. in, 459 adults with a BDI score of at least 14 participated in community-based workshops on self-confidence, using cognitive-behavioural techniques (CBT) addressing emotional components of low self-confidence, cognitive challenges, behavioural methods, and action planning [23]. The intervention significantly reduced depressive symptoms measured at three months post-intervention, with improvements in both primary (BDI) and secondary (GAD-7, RSES) outcomes. Similarly, in a cross-sectional study by Zahra and Saleem involving 394 adolescents, family cohesion was positively correlated with self-confidence and negatively with depression, with self-confidence partially mediating the relationship between family cohesion and depression [24]. Our findings suggest that strengthening self-confidence in ICU survivors could enhance their capacity to cope with cognitive deficits, thereby reducing the risk of depression and supporting long-term recovery.

Given self-confidence’s critical role, our study suggests that interventions specifically designed to enhance self-confidence should be integrated into the post-ICU care plans. Various cognitive-behavioral strategies could effectively address cognitive impairments, improve executive functions, and foster a sense of control and agency, essential for psychological well-being. These interventions have the potential to significantly enhance the quality of life for ICU survivors, helping them regain a positive self-image and better cope with the long-term consequences of their critical illness.

Moreover, our study highlights the need for healthcare providers to recognise the profound impact of the ICU experience on self-confidence and mental health. The extreme stress and disconnection from normal life during ICU stay can lead to a diminished belief in one's ability to overcome challenges, making the reintegration process particularly difficult. Interventions that focus on rebuilding self-confidence are not supplementary but essential for reducing the risk of depression and supporting a successful recovery. Interventions aimed at limiting the disability associated with depression and executive dysfunction, such as problem-solving therapy (PST) have been successfully tested in other, elderly, populations [12]. However, we have found no studies assessing these interventions in a strict ICU survivor population.

This study has several strengths. The high response rate and control for confounders strengthen its internal validity. Structural equation modelling allows for a detailed examination of the mediating role of self-confidence between executive function and depression, increasing methodological rigour [25]. The use of well-validated measures for executive function, self-confidence, and depressive symptoms enhances reliability. Finally, confirmatory factor analysis validates the measurement model, ensuring the robustness of the constructs and their relationships. There are limitations of the study. The cross-sectional design limits our ability to infer causality between executive function, self-confidence, and depressive symptoms, and should be regarded as hypothesis-generating. Future research should employ longitudinal designs to understand the causal directionality of these relationships. Such studies would enable the observation of changes over time and provide clearer insights into how interventions targeting self-confidence influence the trajectory of recovery in cognitive function and mental health outcomes. Moreover, the questions of the provisional questionnaire are mainly based on issues noted by ICU survivors, not on pre-existing questionnaires. Therefore, not all aspects of e.g. the BDI are included, only those raised by the interviewed survivors. Furthermore, the reliance on self-reported measures for executive function, self-confidence, and depressive symptoms may introduce recall bias, which could affect the accuracy of our findings. ICU-specific factors such as length of stay and mechanical ventilation duration were not controlled for in this analysis, and future studies might benefit from examining any potential effect of these factors. Finally, the study sample was drawn from a single university hospital, which may limit the generalisability of our findings to other settings and populations.

## Conclusion

Our study demonstrates that impaired executive function significantly contributes to depressive symptoms in ICU survivors, with self-confidence fully mediating this relationship. While these findings suggest that integrating self-confidence-boosting strategies into post-ICU care may improve ICU survivors’ mental health and quality of life, future longitudinal studies are needed to confirm both the relationships and the long-term effects of such interventions.

## Supplementary Information

Additional file1

## Data Availability

No datasets were generated or analysed during the current study.

## Abbreviations

ADL: Activities of Daily Living
BDI: Beck Depression Inventory
CFI: Comparative Fit Index
FIML: Full Information Maximum Likelihood
HARS: Hamilton Anxiety Rating Scale
ICU: Intensive care unit
LTCI: Long-Term Cognitive Impairment
ML: Maximum Likelihood
PST: Problem-solving therapy
VIF: Variance Inflation Factor
SEM: Structural Equation Modelling
RMSEA: Root Mean Square Error of Approximation
SRMR: Standardized Root Mean Square Residual
TLI: Tucker-Lewis index
